# An In Vitro Study to Assess the Best Strategy for the Chemical Debridement of Periprosthetic Joint Infection

**DOI:** 10.3390/antibiotics12101507

**Published:** 2023-10-02

**Authors:** Miguel Márquez-Gómez, Marta Díaz-Navarro, Andrés Visedo, Rama Hafian, José Matas, Patricia Muñoz, Javier Vaquero, María Guembe, Pablo Sanz-Ruíz

**Affiliations:** 1 Department of Orthopaedic Surgery and Traumatology, Hospital General Universitario Gregorio Marañón, 28007 Madrid, Spain; miguel.marquez@salud.madrid.org (M.M.-G.); joseantonio.matas@salud.madrid.org (J.M.); jvaquero@salud.madrid.org (J.V.); psanzr@salud.madrid.org (P.S.-R.); 2 Instituto de Investigación Sanitaria Gregorio Marañón, 28007 Madrid, Spain; marta.diaz@iisgm.com (M.D.-N.); andres.visedo@iisgm.com (A.V.); pmunoz@hggm.es (P.M.); 3 Department of Clinical Microbiology and Infectious Diseases, Hospital General Universitario Gregorio Marañón, 28007 Madrid, Spain; 4 Faculty of Science, University of Alcalá de Henares, Madrid 28801, Spain; rama.hafian@edu.uah.es; 5 CIBER Enfermedades Respiratorias-CIBERES (CB06/06/0058), 28029 Madrid, Spain; 6 Medicine Department, School of Medicine, Universidad Complutense de Madrid, 28040 Madrid, Spain

**Keywords:** biofilm, stainless implant, in vitro model, antiseptic solutions, irrigation, Bactisure™

## Abstract

Irrigation and debridement using an irrigation solution is a fundamental step during the surgical treatment of both acute and chronic periprosthetic joint infection (PJI). However, there is no consensus on the optimal solution, nor is there sufficient evidence on the optimal irrigation time and combination of solutions. Therefore, it is necessary to determine which solution or combination of solutions is most efficacious against biofilm, as well as the optimal irrigation time. We conducted an experimental in vitro model by inoculating stainless steel discs with ATCC strains of methicillin-susceptible *Staphylococcus aureus*, methicillin-resistant *S. aureus*, *Pseudomonas aeruginosa*, and a clinical strain of *Staphylococcus epidermidis*. The discs were all irrigated with commonly used antiseptic solutions (10% and 3% povidone iodine, hydrogen peroxide, 3% acetic acid, and Bactisure™) for 1 min, 3 min, and 5 min and their combinations for 9 min (3 min each) vs. sterile saline as a positive control. We evaluated the reduction in biofilm based on colony-forming unit (cfu) counts and in combination assays, also based on cell viability and scanning electron microscopy. All antiseptics alone reduced more than 90% of cfu counts after 1 min of irrigation; the worst results were for hydrogen peroxide and 3% acetic acid. When solutions were sequentially combined, the best results were observed for all those starting with acetic acid, in terms of both reduction of log cfu/mL counts and viable cells. We consider that a combination of antiseptic solutions, particularly that comprising the sequence acetic acid + povidone iodine + hydrogen peroxide, would be the best option for chemical debridement during PJI surgery.

## 1. Introduction

Periprosthetic joint infection (PJI) is a severe complication after total knee and hip arthroplasty, with an incidence of 1–2% in primary arthroplasties and up to 4–10% in prosthetic revision surgeries [1]. Despite multiple efforts, PJI is becoming increasingly important owing to the aging of the population, the higher prevalence of comorbidities, and increased life expectancy [2].

According to data from the National Inpatient Sample, a representative sample of all U.S. hospital discharges, the estimated annual incidence of primary total hip and knee arthroplasty per 100,000 persons increased, respectively, by 105% and 119%, between 2000 and 2014 [3]. According to the projection model of Sloan et al., by the year 2030, the volume of total hip arthroplasty is expected to increase by 71.2% to 635,000 procedures, and that of total knee arthroplasty will increase by 84.9% to 1.26 million procedures [3].

PJI not only affects patients, but also places a significant health and economic burden on the system, as many affected patients will undergo multiple surgical procedures and prolonged antibiotic therapy, with a higher cost per treatment [4,5].

PJI is unique, owing to the presence of an implant and its relationship with microorganisms and the immune system. The presence of material means that infection can occur with a much smaller inoculum, up to 100,000 times smaller in the case of *Staphylococcus aureus*, according to some studies, and may be caused by less virulent microorganisms, such as skin saprophytes [6,7]. In addition, bacterial adhesion and biofilm formation are associated with high resistance to antibiotic treatment. In vitro measurement of antibiotic sensitivity does not reflect in vivo sensitivity, as the required concentration cannot be achieved via systemic administration of the antibiotic [8].

The only two treatment options are implant replacement using a one or two-stage procedure, or in selected patients, debridement and antibiotic with implant retention (DAIR) followed by targeted antibiotic treatment [9]. In all these options, chemical debridement through tissue irrigation is a fundamental step, as it decreases bacterial load and rates of re-infection.

The numerous irrigation solutions currently available to orthopedic surgeons range from inexpensive established formulations to expensive commercial antibiofilm formulations. The most commonly used antiseptic solutions for irrigation are 0.35% or 10% povidone iodine, 0.05% chlorhexidine digluconate, 10% hydrogen peroxide, and 3% acetic acid, although none has proven to be superior to the others. While several studies compare the effectiveness of antiseptics on planktonic bacteria, they do not provide sufficient data on the ability of contemporary antiseptic solutions to eradicate biofilm in orthopedic materials [10,11,12]. Only Premkumar et al. [13] observed a clearly superior antibiofilm effect of 10% povidone-iodine after 3 min of exposure, with and without the addition of hydrogen peroxide, against the other solutions studied, such as Bactisure™ (Zimmer Biomet, Madrid, Spain), Irrisept (Irrimax, Orlando, FL, USA), polymyxin-bacitracin, and vancomycin, or Prontosan^®^ diluted solution (B. Braun, Barcelona, Spain). Similarly, the optimal irrigation time and combination of antiseptics have not been addressed [14,15,16].

We attempted to answer these questions by performing an experimental in vitro study to determine which solution showed the best antibiofilm activity, how long it took to achieve its effect, and whether sequential combinations of solutions improved results.

## 2. Results

### 2.1. Single Antiseptics

**Figure 1:**
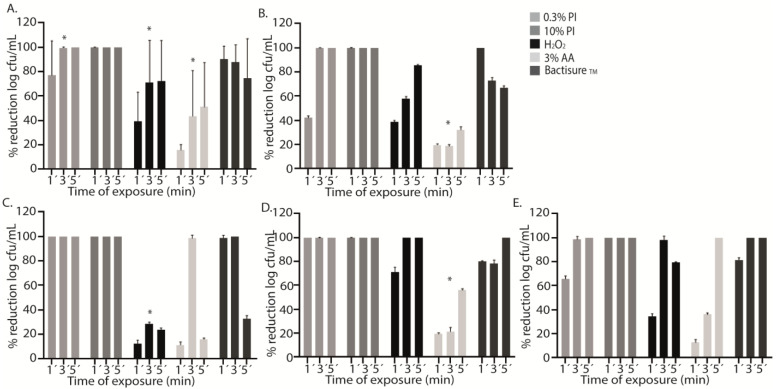
Graphical representation of the efficacy of different antiseptics against each microorganism. The graphs show the mean percentage reduction in log cfu/mL of each antiseptic (0.3% PI, 0.3% povidone iodine; 10% PI, 10% povidone iodine; H_2_O_2_, hydrogen peroxide; AA3, 3% acetic acid, and Bactisure™) in all microorganisms (**A**) and for each species studied separately: methicillin-susceptible *Staphylococcus aureus* (MSSA) (**B**), methicillin-resistant *Staphylococcus aureus* (MRSA) (**C**), *Staphylococcus epidermidis* (**D**), and *Pseudomonas aeruginosa* (**E**) at three different exposure times (1′, 3′, and 5′). The differences between log cfu/mL reduction at different exposure times for each antiseptic were statistically significant (* *p* < 0.001) for 0.3% PI, H_2_O_2_, and AA3 (**A**). AA showed a lower bacterial decrease at the three exposure times (* *p* < 0.001) (**B**). H_2_O_2_ showed a lower bacterial decrease than 0.3% PI, 10% PI, and Bactisure™ (* *p* < 0.001) (**C**). AA3 showed a lower bacterial decrease at the three exposure times (* *p* < 0.001) (**D**).

Compared to the positive control, a statistically significant decrease in the median (IQR) log cfu/mL was observed for all antiseptics at all exposure times (1, 3, and 5 min) (Table 1, Figure 1A). However, the decrease was less notable with AA3 at all exposure times, *p* < 0.001 (Table 1, Figure 1A).

In PI treatment, we did not obtain any cfu from the sonicated discs after 3 and 5 min of exposure, whereas after 1 min of exposure, the mean (SD) percentage reduction reached only 77.1% (12.0%). As with PI, no bacteria were recovered from sonicated discs after PI10 treatment, regardless of exposure time (Table 1, Figure 1A).

In contrast, treatment with H_2_O_2_, AA3, and Bactisure™ did not reach 100% reduction in cfu counts. The mean (SD) percentage reduction in log cfu/mL after 1 min, 3 min, and 5 min with H_2_O_2_ was, respectively, 39.0% (22.1%), 71.1% (32.1%), and 72.1% (29.7%). The mean (SD) percentage reduction in log cfu/mL after 1 min, 3 min, and 5 min with AA3 was, respectively, 15.7% (4.4%), 43.9% (34.7%), and 51.2% (33.0%). The mean (SD) percentage reduction in log cfu/mL after 1 min, 3 min, and 5 min with Bactisure™ was, respectively, 91.0% (9.5%), 87.8% (13.0%), and 74.5% (29.4%) (Table 1, Figure 1A).

H_2_O_2_, AA3, and Bactisure™ reached a significantly lower bacterial decrease at all exposure times compared to PI and PI10 in terms of median log cfu/mL count (Table 2).

Analysis of the effect of antiseptic solutions on specific microorganisms showed a mean (SD) percentage reduction in log cfu/mL for AA3 of only 23.3% (6.0%) (*p* < 0.001), with a lower bacterial decrease in MSSA and *S. epidermidis* at the three exposure times, reaching a mean (SD) percentage reduction of 31.2% (5.7%) and 56.6% (7.1%) after 5 min, respectively (*p* < 0.001) (Figure 1B,D). However, in *P. aeruginosa* and MRSA, complete bacterial eradication was reached at 5 min and 3 min, respectively (Figure 1C,E). H_2_O_2_ showed a lower bacterial decrease in MRSA than PI, PI10, and Bactisure™ (*p* < 0.001) (Table 2, Figure 1C).

### 2.2. Sequential Combination of Antiseptics

**Figure 2:**
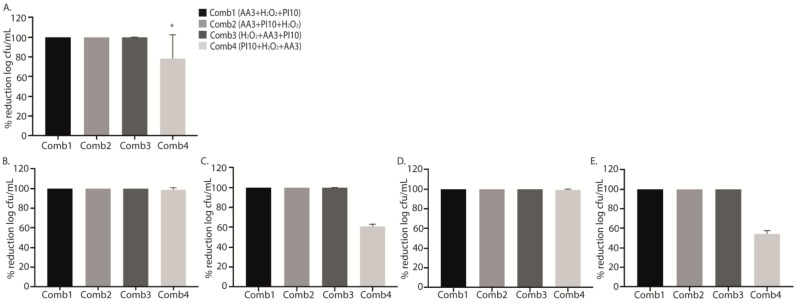
Graphical representation of the efficacy of the four sequential antiseptic combinations against each microorganism in terms of percentage reduction in log cfu/mL. Data show the mean percentage reduction in log cfu/mL for each sequential antiseptic combination applied against all microorganisms (**A**) and for each microorganism separately: methicillin-susceptible *Staphylococcus aureus* (MSSA) (**B**), methicillin-resistant *Staphylococcus aureus* (MRSA) (**C**), *Staphylococcus epidermidis* (**D**), and *Pseudomonas aeruginosa* (**E**) at a single exposure time of 3 min. The decrease in bacterial concentration for the sequential combination 4 (PI10 + H_2_O_2_ + AA3) was lower than for the other sequential combinations (* *p* = 0.006, in all comparisons) (**A**). No statistically significant differences were found according to species. **Comb1**, sequential combination 1; **Comb2**, sequential combination 2; **Comb3**, sequential combination 3; **Comb4**, sequential combination; **PI**, povidone iodine; **H_2_O_2_**, hydrogen peroxide; **AA3**, acetic acid.

Sequential washing for 9 min (3 min with each solution) with the sequential combinations 1, 2 and 3 (AA3 + H_2_O_2_ + PI10, AA3 + PI10 + H_2_O_2_, and H_2_O_2_ + AA3 + PI10) resulted in sterile cultures after sonication for all strains, whereas the sequential combination 4 (PI10 + H_2_O_2_ + AA3) only reached a log cfu/mL percentage reduction of 78.8%, *p* = 0.006, being less active for MRSA and *P. aeruginosa* (61.3% and 53.7%, respectively) (Table 3, Figure 2A,C,E). However, in MSSA and *S. epidermidis*, complete bacterial eradication was achieved (Figure 2B,D).

**Figure 3:**
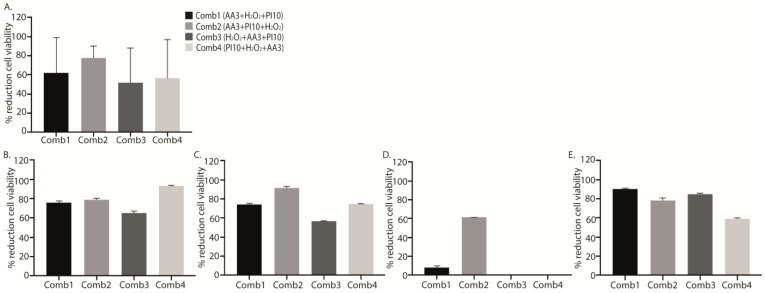
Graphical representation of the efficacy of the four sequential antiseptic combinations against each microorganism in terms of percentage reduction in log cfu/mL. Data show the mean percentage reduction in log cfu/mL for each sequential antiseptic combination applied against all microorganisms (**A**) and for each microorganism separately: methicillin-susceptible *Staphylococcus aureus* (MSSA) (**B**), methicillin-resistant *Staphylococcus aureus* (MRSA) (**C**), *Staphylococcus epidermidis* (**D**), and *Pseudomonas aeruginosa* (**E**) at a single exposure time of 3 min. No statistically significant differences were found in these analysis. **Comb1**, sequential combination 1; **Comb2**, sequential combination 2; **Comb3**, sequential combination 3; **Comb4**, sequential combination 4; **PI**, povidone iodine; **H_2_O_2_**, hydrogenated peroxide; **AA3**, acetic acid.

Although no significant differences were found, all data obtained from the treatments with the different sequential combinations showed a marked reduction in cell viability with respect to positive controls, with the mean (SD) percentage reduction for sequential combinations 1, 2, 3 and 4 (AA3 + H_2_O_2_ + PI10, AA3 + PI10 + H_2_O_2_, H_2_O_2_ + AA3 + PI10, and PI10 + H_2_O_2_ + AA3) being, respectively, 62.28 ± 33.26%, 77.63 ± 11.40%, 51.68 ± 32.99%, and 56.70 ± 36.43% (*p* = 0.103) (Table 4, Figure 3).

**Figure 4:**
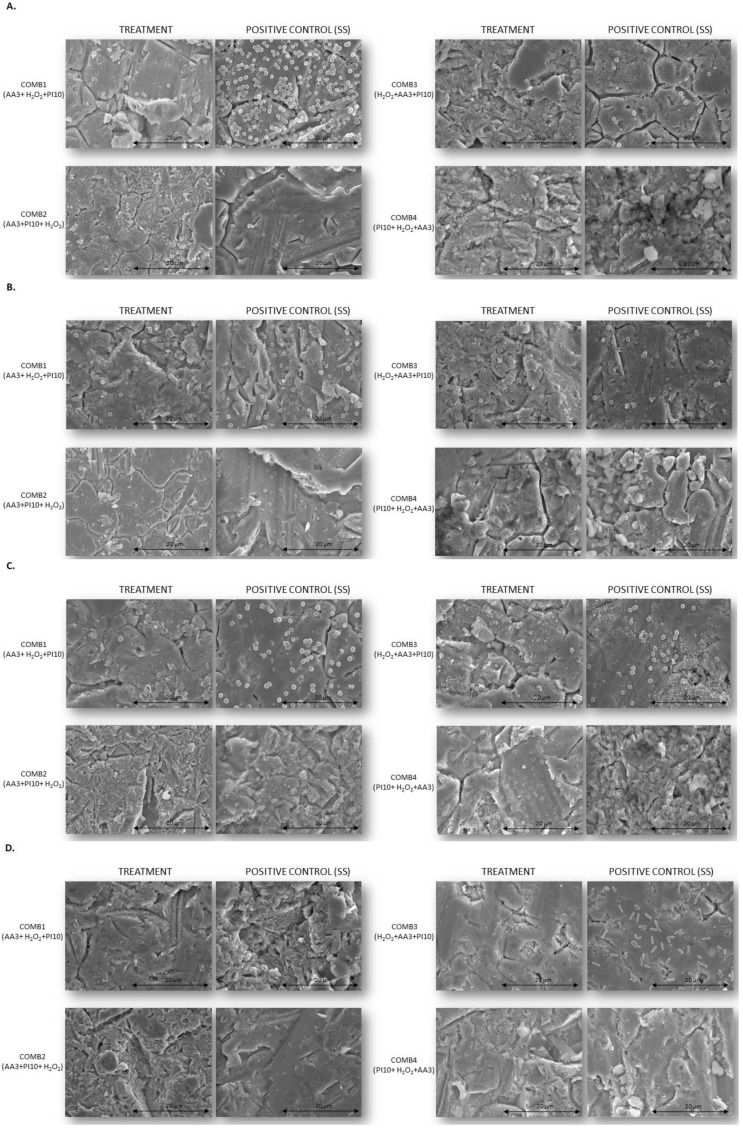
SEM images of biofilms treated with antiseptic combinations at a single exposure time of 3 min each vs. positive controls treated with sterile saline. All images were taken at 2500× magnification. (**A**). Methicillin-susceptible *Staphylococcus aureus*; (**B**). Methicillin-resistant *Staphylococcus aureus*; (**C**). *Staphylococcus epidermidis*; (**D**). *Pseudomonas aeruginosa*. **Comb1**, sequential combination 1; **Comb2**, sequential combination 2; **Comb3**, sequential combination 3; **Comb4**, sequential combination 4; **PI**, povidone iodine; **H_2_O_2_**, hydrogenated peroxide; **AA3**, acetic acid.

The results obtained were confirmed by the SEM images (Figure 4).

## 3. Discussion

Surgical and chemical debridement are key steps in the surgical treatment of PJI. They serve to decrease the bacterial load and rate of infection. However, there is no consensus on which antiseptic solution to use and for how long it should be applied. In fact, delegates at the 2018 International Consensus Meeting on Musculoskeletal Infection did not vote in favor of using antiseptics routinely in primary arthroplasty, although they did vote strongly in favor of using antiseptics during surgery for PJI, despite scarce evidence of their efficacy [17].

Previous in vitro studies reported that exposure times between 1 and 5 min were sufficient for the antiseptics to be effective and do not excessively increase operative time [13,18,19]. However, based on our results, 1 min did not lead to complete bacterial eradication in several experiments.

Regarding the efficacy of each antiseptic when used individually, 0.3% PI applied for at least 3 min may be the best choice, owing to its lower toxicity, 1 min application with PI10 would be also sufficient. Our data are consistent with those of other, previous studies that tested PI. Shohat et al. analyzed 31,331 cases of PJI, in which 0.3% PI was used for 3 min, and found the rate of PJI to be 2.34 times lower than for the control group (0.6% vs. 1.3%, *p* < 0.001) [20]. Oduwole et al. studied the effect of PI on *S. aureus* and *S. epidermidis*, reporting that in addition to its known bactericidal effect, it can also inhibit the development of *S. epidermidis* and *S. aureus* biofilm by repressing transcription of polysaccharide intercellular adhesin [18]. Ernest et al. studied the effect of PI10 on cobalt chrome, stainless steel, and titanium alloy discs with MSSA biofilm and found that irrigation for 5 min reduces biofilm growth by 95%, and that this option was superior to H_2_O_2_, ClO_2_, and Dakin’s solution [19]. Premkumar et al. analyzed the anti-biofilm activity of several antiseptic solutions on different surfaces of common implants such as PMMA and polyethylene, and found that PI10 and its combination with 4% H_2_O_2_ were the most effective, although the concentrations are considered cytotoxic [13]. One of the concerns associated with antiseptics in vivo is soft tissue toxicity at the concentration used, and the possibility of inhibiting tissue healing. Romano et al. studied the cytotoxicity of 0.3%PI, 0.5% PI, 0.5% H_2_O_2_, and 1.5% H_2_O_2_ after 1, 3, and 5 min of exposure, reporting a gradual increase in tissue toxicity according to exposure time, except for 0.3% PI, for which the lowest toxicity was recorded [21]. Therefore, we consider that PI0.3 could be used for prophylaxis, rather than PI10, whose toxicity in soft tissues is still a concern. However, all toxicity studies were performed in vitro and examined antiseptics used for prophylactic purposes (healthy joint). These results have not been validated in the context of PJI, and there is no evidence that PI0.3 3 min is more toxic than PI10 1 min. Our in vitro studies lead us to believe that it could be.

Bactisure™ is a commercialized irrigation solution that includes ethanol, acetic acid, sodium acetate, and benzalkonium chloride. Its anti-biofilm activity has previously been tested by Kia et al., who reported no differences in the reduction in bacterial load of *S. aureus* compared to PI [15]. We recorded better results for PI than for Bactisure™ after 3 and 5 min of irrigation and PI10 at all exposure times.

While combining solutions may have a synergistic effect, not all solutions can be combined [22], as 4% chlorhexidine gluconate precipitates with PI and H_2_O_2_, and there is no evidence of its anti-biofilm activity; therefore, it is not recommended in combinations. In addition, 0.5% sodium hypochlorite generates gas when combined with PI or H_2_O_2_, and the combination of AA and H_2_O_2_ can form peracetic acid. This last interaction does not affect our results, since, during the combinations, the 3 min sequential exposure with each of the different solutions is always followed by a saline wash to rinse the previous solution. Therefore, the only antiseptics that do not react with each other are PI and H_2_O_2_. In fact, in their in vitro study, Premkumar et al. showed that H_2_O_2_ had a synergistic effect with PI, their combination being the most effective [13].

In our in vitro study, the highest anti-biofilm activity was achieved when antiseptics were combined. In addition, the sequential order in which each antiseptic was applied affected the results, as the only sequential combination that did not reach complete bacterial eradication was that in which AA3 was not used in the first step. Therefore, since AA3 disrupts the biofilm, we believe that it should be used at the beginning, followed by PI and H_2_O_2_, which both have a bactericidal effect (sequential combination 2).

We believe that specific triple treatment is a key factor in chemical debridement during surgery for PJI. In some in vitro studies, an increase in the antibacterial effect of PI was observed after the application of H_2_O_2_; therefore, it can be stated that H_2_O_2_ has a synergistic effect with iodine. This effect was not observed when iodine was used in isolation. However, in highly mature biofilm, the sequential combination of H_2_O_2_ and PI might not be 100% effective. The use of acetic acid prior to this sequential combination is based on its ability to alter the polysaccharide matrix as an acidic liquid, not on its antiseptic effect, as it needs prolonged exposure to show this effect. In line with the mechanism of action of AA3, our in vitro study revealed this effect when we used AA3 at the beginning of the sequential combination, but not when it was used after other antiseptic solutions.

Although we observed clear statistically significant differences according to the reduction in cfu counts, we were not able to confirm these results with respect to cell viability using flow cytometry, because samples had been refrigerated for several days, thus potentially affecting viability. In addition, the lack of correlation between percentage reduction in cfu counts and cell viability rate could be due to the presence of viable but nonculturable cells (VBNC), as we previously demonstrated with breast prostheses [23]. Therefore, it is important to analyze not only cfu counts, but also cell viability, as the presence of VBNC may have an important clinical impact.

One of the main limitations of the study was that it was based on a 24 h in vitro biofilm model, which may not mimic a real clinical scenario in which mature biofilms are formed [16]. In this regard, it is complicated to observe differences between high-efficacy solutions against early biofilm, as occurred in our study, given the high efficacy of PI10 and PI0.3. However, our conclusions remain unaffected: of the individual solutions, PI10 is the best, as it achieves 100% efficacy in less time (1 min). In addition, of the sequential combinations, AA3 + H_2_O_2_ + PI10 is the best; starting with AA3 enables biofilm disruption followed by the synergistic antiseptic action of H_2_O_2_ and PI.

Another limitation of the study is that the samples were refrigerated, potentially affecting cell viability, since higher cell viability values were expected in the positive controls. In addition, the mechanical effect of pulsed washing was not evaluated, since we performed the irrigation via immersion of the steel disc in the solutions tested. Moreover, since we only studied the antibiofilm activity of antiseptics on stainless steel implants, the results may differ depending on the material, as previously demonstrated [13]. It seems that the biofilm does not grow equally on all surfaces (plastic > titanium > PMMA), thus potentially influencing the antibiofilm capacity of the antiseptic solutions tested. Therefore, it would be interesting to consider this in future in vitro models. We did not test other irrigation solutions, such as surfactants, because they have no bactericidal effect and lead to regrowth, and in animal models, they failed to show better antibiophilic activity than saline [11]. Similarly, topical antibiotics were not evaluated, as there is evidence that their use does not improve the results after DAIR, with inadequate eradication rates reported for *Staphylococcus* spp. and *E. coli* [24]. Lastly, SEM studies should have been performed after each treatment step in the combinations to confirm the cumulative effect. However, this analysis is very time-consuming and expensive.

Therefore, our data should be validated with other materials, and further clinical data are necessary to determine whether these solutions can reduce PJI in in vivo models.

## 4. Materials and Methods

### 4.1. Setting

The study was performed in the microbiology laboratory of a tertiary institution in Madrid, Spain.

### 4.2. Laboratory Procedure

We designed an in vitro model with stainless steel disc implants based on a two-phase model with a contamination step (24 h biofilm), in which each strain was tested before being disinfected with antiseptics and their combinations (detailed below) or sterile saline (SS, for positive controls). Different exposure times were chosen to assess the impact on colony-forming unit (cfu) counts and, in combination assays, on cell viability and biofilm structure.

#### 4.2.1. Strains

We selected three ATCC strains, namely, ATCC29213 methicillin-susceptible *S. aureus*, ATCC43300 methicillin-resistant *S. aureus*, and ATCC15442 *Pseudomonas aeruginosa*. We also selected a clinical strain of *Staphylococcus epidermidis* showing optimal biofilm production (by crystal violet assay) from the microbiology laboratory, and a corresponding to the MICRO.HGUGM-2016-027 project.

#### 4.2.2. Antiseptics

- 0.3% povidone iodine (PI0.3)
- 10% povidone iodine (PI10)
- Hydrogen peroxide (H_2_O_2_)
- 3% acetic acid (AA3)
- Bactisure™ (ethanol [solvent], acetic acid [pH modifier], sodium acetate [buffer], benzalkonium chloride [surfactant], and water)

#### 4.2.3. Sequential Combinations

- Sequential combination 1, AA3 + H_2_O_2_ + PI10 (Comb1).
- Sequential combination 2, AA3 + PI10 + H_2_O_2_ (Comb2).
- Sequential combination 3, H_2_O_2_ + AA3 + PI10 (Comb3).
- Sequential combination 4, PI10 + H_2_O_2_ + AA3 (Comb4).

Stainless steel discs measuring 6 mm in diameter and 3 mm in height were prepared by the ICAI School of Engineering, Pontificia Comillas University. The discs were sterilized using ethanol immersion followed by autoclaving (121 °C, 15 min) before use.

#### 4.2.4. Methodology

**Figure 5:**
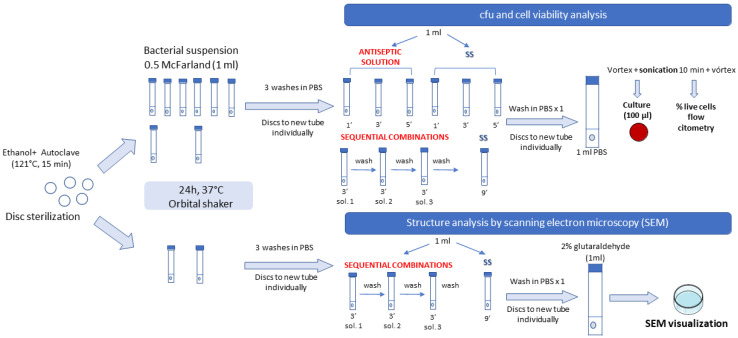
Laboratory procedure. Schematic diagram of the laboratory procedure followed for the analysis. **SS**, sterile saline, **PBS**, phosphate buffered saline; **cfu**, colony forming units; **SEM**, scanning electron microscopy.

The two-phase model was based on a contamination step, in which discs were immersed in glass tubes at a ratio of 1 disc/1 mL of phosphate-buffered saline (PBS), followed by gentle washing to remove nonadherent bacteria. The discs were then disinfected by applying each of the antiseptics at 1 min, 3 min, and 5 min, with 9 min for each combination assay (3 min per antiseptic). Positive controls were treated the same with SS. Next, gentle washing was performed, followed by sonication in 1 mL of PBS at 40 kHz for 10 min (Figure 5).

All experiments were carried out in triplicate.

Contamination step: discs were placed into glass tubes containing 1 mL of bacterial suspension (0.5 McFarland = 2.5 × 10^8^ cfu/mL) of each strain in PBS. The negative control was inoculated with only PBS. Tubes were incubated in an orbital shaker at 37 °C for 24 h. After this period, discs were washed three times with PBS to remove nonadherent bacteria.

Disinfection step: discs were placed into glass tubes containing 1 mL of each antiseptic solution for either 1 min, 3 min, or 5 min. For sequential combination experiments of three antiseptic solutions, only 3 min of exposure time was assessed for each solution vs. 9 min of SS. The same procedure was carried out in parallel with 0.9% SS as a positive control. The negative control was treated with PI for 1 min. The model was performed in order to mimic a clinical scenario; therefore, in the disinfection step, the contaminated discs were transferred to the antiseptic solution (or SS in positive controls), considering that the antiseptics act during the disinfection step for the different exposure times.

Sessile cell recovery: After washing, the discs were individually transferred to new glass tubes containing 1 mL PBS, and sonicated for 10 min at 40 kHz to detach the biofilm. The sonicated bacterial suspension was then vortexed for culture.

Culture of sonicate: Once the sonicated bacterial suspension had been vortexed, one part was serially diluted, and 100 µL of dilution was cultured on blood agar plates. Plates were incubated at 37 °C for 24 h and cfu/plate counts were calculated and expressed on a logarithmic scale.

Cell viability rate: In the sequential combination assays, the sonicated bacterial suspensions were refrigerated and further stained on slides with the commercial kit BacLight^®^ (ThermoFisher Scientific, Madrid, Spain) (comprising Syto 9, which stains living cells green, and propidium iodide, which stains damaged cells red) according to the manufacturer’s instructions. The samples were analyzed using flow cytometry (Gallios, Beckman Coulter, BioRad, Madrid, Spain) and the images were acquired by adjusting the delimitations to include only singlets in the analysis. The resulting data were analyzed using the Kaluza software application.

The experiments were performed in the same way for the biofilm structure, although using one disc per strain and treatment. After the disinfection step, discs were directly and individually transferred to a new glass tube containing 1 mL of 2% glutaraldehyde for subsequent visualization of biofilm thickness via scanning electron microscopy (SEM) (three fields in each disc). For observation of biofilm structure using SEM, samples were dehydrated with increasing concentrations of 30%, 50%, 70%, 80%, and 90% ethanol for 10 min and absolute ethanol for 20 min. Differences in the structure and occupancy of the biofilms formed were analyzed using a 2500× objective (JEOL 6400 JSM, Labexchange, Burladingen, Germany).

#### 4.2.5. Statistical Analysis

Quantitative variables are expressed as the median and interquartile range (IQR).

The median (IQR) and mean (SD) log cfu/mL were calculated for each experiment. In sequential combination assays, the median (IQR) and mean (SD) cell viability rates were also analyzed. Median (IQR) and mean (SD) values are detailed in the tables and figures, respectively. The interquartile range includes all values falling within the 25th and 75th percentiles.

For comparisons between groups, we used parametric methods (a *t* test or ANOVA) or nonparametric methods (a median test or Kruskal–Wallis test). Linear or logistic regression models were fitted in cases of asymmetry.

Statistical significance was set at *p* < 0.05 for all the tests. The statistical analysis was performed using IBM SPSS Statistics for Windows, Version 21.0 (IBM Corp, Armonk, NY, USA) and Software GraphPad Prism 8.0 (San Diego, CA, USA) (accessed on 5 June 2023).

### 4.3. Availability of Data and Material

Datasets will be kept by the Microbiology and Infectious Diseases Department, and data collection will be registered in the repository of the Instituto de Salud Carlos III (ISCIII) under number C.0001228.

## 5. Conclusions

Based on our results, we propose a sequential combination of 9 min exposure of AA3 + PI10 + H_2_O_2_ (sequential combination 2). AA3 ensures chemical debridement and disruption of the biofilm, followed PI10, which has a bactericidal effect, then followed by H_2_O_2_, which has a bactericidal effect and increases the porosity of the cell wall. However, when sequential combinations are not available, PI may be a cost-effective alternative, as its anti-biofilm efficacy outperformed that of all the other solutions.

## Figures and Tables

**Table 1:** Results for antiseptic at three exposure times.

Antiseptic Solution	Treatment	Median (IQR) log cfu/mL	*p* *
PI0.3	SS 1′	5.56 (5.13–6.05)	**<0.001**
	PI0.3 1′	1.08 (0.00–3.01)	
	SS 3′	5.64 (4.86–5.98)	**<0.001**
	PI0.3 3′	0.00 (0.00–0.00)	
	SS 5′	5.64 (5.36–6.05)	**<0.001**
	PI0.3 5′	0.00 (0.00–0.00)	
PI10	SS 1′	4.99 (4.08–5.49)	**<0.001**
	PI10 1′	0.00 (0.00–0.00)	
	SS 3′	5.17 (4.65–5.72)	**<0.001**
	PI10 3′	0.00 (0.00–0.00)	
	SS 5′	5.51 (4.55–5.85)	**<0.001**
	PI10 5′	0.00 (0.00–0.00)	
H_2_O_2_	SS 1′	5.69 (4.97–6.53)	**<0.001**
	H_2_O_2_ 1′	4.08 (2.13–4.29)	
	SS 3′	5.95 (5.01–7.04)	**<0.001**
	H_2_O_2_ 3′	1.40 (0.00–3.38)	
	SS 5′	5.78 (4.84–6.94)	**<0.001**
	H_2_O_2_ 5′	1.24 (0.25–3.07)	
AA3	SS 1′	5.59 (5.11–6.34)	**0.002**
	AA3 1′	4.09 (4.23–5.45)	
	SS 3′	5.28 (4.78–6.1)	**<0.001**
	AA3 3′	3.86 (0.93–4.36)	
	SS 5′	5.56 (5.21–6.40)	**<0.001**
	AA3 5′	3.15 (0.58–4.23)	
Bactisure^TM^	SS 1′	3.84 (5.21–6.40)	**<0.001**
	Bactisure^TM^ 1′	0.50 (0.00–1.00)	
	SS 3′	6.12 (5.27–6.42)	**<0.001**
	Bactisure^TM^ 3′	0.50 (0.00–1.59)	
	SS 5′	6.18 (5.45–6.48)	**<0.001**
	Bactisure^TM^ 5′	1.10 (0.00–3.55)	

**SS**, sterile saline; **PI**, povidone iodine; **H_2_O_2_**, hydrogen peroxide; **AA3**, acetic acid; **IQR**, interquartile range; **cfu**, colony-forming units. ***p***, *p* value, * Statistically significant values are shown in bold.

**Table 2:** Comparison between each antiseptic solution at each exposure time.

	Treatment	Median (IQR) log cfu/mL	*p* *	Median (IQR) % Live Cells	*p* *
PI0.3 vs. PI10	PI0.3 1′	1.08 (0.00–3.01)	**0.006**	NA	NA
	PI10 1′	0.00 (0.00–0.00)			
	PI0.3 3′	0.00 (0.00–0.00)	NA		
	PI10 3′	0.00 (0.00–0.00)			
	PI0.3 5′	0.00 (0.00–0.00)	NA		
	PI10 5′	0.00 (0.00–0.00)			
PI0.3 vs. H_2_O_2_	PI0.3 1′	1.08 (0.00–3.01)	**<0.001**	NA	NA
	H_2_O_2_ 1′	4.08 (2.13–4.29)			
	PI0.3 3′	0.00 (0.00–0.00)	**0.006**		
	H_2_O_2_ 3′	1.40 (0.00–3.38)			
	PI0.3 5′	0.00 (0.00–0.00)	**<0.001**		
	H_2_O_2_ 5′	1.24 (0.25–3.07)			
PI0.3 vs. AA3	PI0.3 1′	1.08 (0.00–3.01)	**0.001**	NA	NA
	AA3 1′	4.09 (4.23–5.45)			
	PI0.3 3′	0.00 (0.00–0.00)	**<0.001**		
	AA3 3′	3.86 (0.93–4.36)			
	PI0.3 5′	0.00 (0.00–0.00)	**<0.001**		
	AA3 5′	3.15 (0.58–4.23)			
PI0.3 vs. Bactisure^TM^	PI03 1′	1.08 (0.00–3.01)	0.262	NA	NA
	Bactisure™ 1′	0.50 (0.00–1.00)			
	PI0.3 3′	0.00 (0.00–0.00)	**0.006**		
	Bactisure™ 3′	0.50 (0.00–1.59)			
	PI0.3 5′	0.00 (0.00–0.00)	**0.002**		
	Bactisure™ 5′	1.10 (0.00–3.55)			
PI10 vs. H_2_O_2_	PI10 1′	0.00 (0.00–0.00)	**<0.001**	NA	NA
	H_2_O_2_ 1′	4.08 (2.13–4.29)			
	PI10 3′	0.00 (0.00–0.00)	**0.006**		
	H_2_O_2_ 3′	1.40 (0.00–3.38)			
	PI10 5′	0.00 (0.00–0.00)	**<0.001**		
	H_2_O_2_ 5′	1.24 (0.25–3.07)			
PI0.3 vs. AA3	PI10 1′	0.00 (0.00–0.00)	**<0.001**	NA	NA
	AA3 1′	4.09 (4.23–5.45)			
	PI10 3′	0.00 (0.00–0.00)	**<0.001**		
	AA3 3′	3.86 (0.93–4.36)			
	PI10 5′	0.00 (0.00–0.00)	**<0.001**		
	AA3 5′	3.15 (0.58–4.23)			
PI10 vs. Bactisure^TM^	PI10 1′	0.00 (0.00–0.00)	**0.006**	NA	NA
	Bactisure™ 1′	0.50 (0.00–1.00)			
	PI10 3′	0.00 (0.00–0.00)	**0.006**		
	Bactisure™ 3′	0.50 (0.00–1.59)			
	PI10 5′	0.00 (0.00–0.00)	**0.006**		
	Bactisure™ 5′	1.10 (0.00–3.55)			
H_2_O_2_ vs. AA3	H_2_O_2_ 1′	4.08 (2.13–4.29)	**<0.001**	NA	NA
	AA3 1′	4.09 (4.23–5.45)			
	H_2_O_2_ 3′	1.40 (0.00–3.38)	**0.007**		
	AA3 3′	3.86 (0.93–4.36)			
	H_2_O_2_ 5′	1.24 (0.25–3.07)	0.065		
	AA3 5′	3.15 (0.58–4.23)			
H_2_O_2_ vs. Bactisure^TM^	H_2_O_2_ 1′	4.08 (2.13–4.29)	**<0.001**	NA	NA
	Bactisure™ 1′	0.50 (0.00–1.00)			
	H_2_O_2_ 3′	1.40 (0.00–3.38)	0.265		
	Bactisure™ 3′	0.50 (0.00–1.59)			
	H_2_O_2_ 5′	1.24 (0.25–3.07)	1.000		
	Bactisure™ 5′	1.10 (0.00–3.55)			
AA3 vs. Bactisure^TM^	AA3 1′	4.09 (4.23–5.45)	**<0.001**	NA	NA
	Bactisure™ 1′	0.50 (0.00–1.00)			
	AA3 3′	3.86 (0.93–4.36)	**0.007**		
	Bactisure™ 3′	0.50 (0.00–1.59)			
	AA3 5′	3.15 (0.58–4.23)	0.059		
	Bactisure™ 5′	1.10 (0.00–3.55)			

**PI**, povidone iodine; **H_2_O_2_**, hydrogen peroxide; **AA3**, acetic acid; **IQR**, interquartile range; **cfu**, colony-forming units, **NA**, not applicable; ***p***, *p* value. * Statistically significant values are shown in bold.

**Table 3:** Results for sequential antiseptic combinations at a single exposure time of 3 min each.

Sequential Combination	Treatment	Median (IQR) log cfu/mL	*p* *	Median (IQR) % Live Cells	*p* *
Comb1 vs. Comb2	AA3 3′+ H_2_O_2_ 3′+PI10 3′	0.00 (0.00–0.00) 0.00 (0.00–0.00)	NA	10.89 (4.03–16.97) 6.04 (3.90–8.60)	0.248
	AA3 3′+PI10 3′+ H_2_O_2_ 3′				
Comb1 vs. Comb3	AA3 3′+ H_2_O_2_ 3′+PI10 3′	0.00 (0.00–0.00) 0.00 (0.00–0.00)	NA	10.89 (4.03–16.97) 16.89 (5.87–32.49)	0.386
	H_2_O_2_ 3′+AA3 3′+PI10 3′				
Comb1 vs. Comb4	AA3 3′+ H_2_O_2_ 3′+PI10 3′	0.00 (0.00–0.00) 1.19 (0.00–2.67)	**0.006**	10.89 (4.03–16.97) 9.35 (4.04–26.85)	0.773
	PI10 3′+ H_2_O_2_ 3′+AA3 3′				
Comb2 vs. Comb3	AA3 3′+PI10 3′+ H_2_O_2_ 3′	0.00 (0.00–0.00) 0.00 (0.00–0.00)	NA	6.04 (3.90–8.60) 16.89 (5.87–32.49)	0.248
	H_2_O_2_ 3′+AA3 3′+PI10 3′				
Comb2 vs. Comb4	AA3 3′+PI10 3′+ H2O2 3′	0.00 (0.00–0.00) 1.19 (0.00–2.67)	**0.006**	6.04 (3.90–8.60) 9.35 (4.04–26.85)	0.564
	PI10 3′+ H_2_O_2_ 3′+AA3 3′				
Comb3 vs. Comb4	H_2_O_2_ 3′+AA3 3′+PI10 3′	0.00 (0.00–0.00)	**0.006**	16.89 (5.87–32.49)	0.468
	PI10 3′+ H_2_O_2_ 3′+AA3 3′				

**PI**, povidone iodine; **H_2_O_2_**, hydrogen peroxide; **AA3**, acetic acid; **IQR**, interquartile range; **cfu**, colony-forming units. * Statistically significant values are shown in bold. **Comb1**, sequential combination 1; **Comb2**, sequential combination 2; **Comb3**, sequential combination 3; **Comb4**, sequential combination 4. **NA**, not applicable; ***p****, p* value.

**Table 4:** Results for antiseptic combinations at a single exposure time of 3 min each.

	Treatment	Median (IQR) log cfu/mL	*p* *	Median (IQR) % Live Cells	*p* *	Median (IQR) % Reduction Live Cells ^1^	*p* *
	SS 9′	6.43 (6.39–6.45)	**<0.001**	32.20 (19.29–43.74)	0.042	75.00 (25.63–87.00)	0.103
Comb1	AA3 3′ + H_2_O_2_ 3′ + PI10 3′	0.00 (0.00–0.00)		10.89 (4.03–16.97)			
	SS 9′	5.96 (5.48–6.10)	**<0.001**	32.20 (19.29–43.74)	0.020	79.75 (65.08–87.95)	
Comb2	AA3 3′ + PI10 3′ + H_2_O_2_ 3′	0.00 (0.00–0.00)		6.04 (3.90–8.60)			
	SS 9′	5.08 (5.08–5.08)	**<0.001**	32.20 (19.29–43.74)	0.081	59.90 (13.95–79.70)	
Comb3	H_2_O_2_ 3′ + AA3 3′ + PI10 3′	0.00 (0.00–0.00)		16.89 (5.87–32.49)			
	SS 9′	6.40 (6.01–6.62)	**<0.001**	32.20 (19.29–43.74)	0.081	67.10 (14.50–87.75)	
Comb4	PI10 3′ + H_2_O_2_ 3′ + AA3 3′	1.19 (0.00–2.67)		9.35 (4.04–26.85)			

**PI**, povidone iodine; **H_2_O_2_**, hydrogenated peroxide; **AA3**, acetic acid; **IQR**, interquartile range; **cfu**, colony-forming units; **Comb1**, sequential combination 1; **Comb2**, sequential combination 2; **Comb3**, sequential combination 3; **Comb4**, sequential combination 4; **SS**; sterile saline. ^1^ Data represent the percentage of live cells reduction for each sequential antiseptic combination. ***p****, p* value, * Statistically significant values are shown in bold.

## Data Availability

Not applicable.
